# Composite Behavior of a Novel Insulated Concrete Sandwich Wall Panel Reinforced with GFRP Shear Grids: Effects of Insulation Types

**DOI:** 10.3390/ma8030899

**Published:** 2015-03-03

**Authors:** JunHee Kim, Young-Chan You

**Affiliations:** 1 Department of Architectural Engineering, Yonsei University, 50 Yonseiro, Seodaemun-gu, Seoul 120-749, Korea; 2 Department of Building Research, Korea Institute of Civil Engineering and Building Technology, 283 Goyangdae-ro, Gogang-si, Gyeonggi-do 411-712, Korea; E-Mail: ycyou@kict.re.kr

**Keywords:** glass-fiber-reinforced polymer (GFRP) shear grids, insulated concrete sandwich wall panels, flexural strength, composite action, insulation effect

## Abstract

A full-scale experimental program was used in this study to investigate the structural behavior of novel insulated concrete sandwich wall panels (SWPs) reinforced with grid-type glass-fiber-reinforced polymer (GFRP) shear connectors. Two kinds of insulation-expanded polystyrene (EPS) and extruded polystyrene (XPS) with 100 mm thickness were incased between the two concrete wythes to meet the increasing demand for the insulation performance of building envelope. One to four GFRP shear grids were used to examine the degree of composite action of the two concrete wythes. Ten specimens of SWPs were tested under displacement control subjected to four-point concentrated loads. The test results showed that the SWPs reinforced with GFRP grids as shear connectors developed a high degree of composite action resulting in high flexural strength. The specimens with EPS foam exhibited an enhanced load-displacement behavior compared with the specimens with XPS because of the relatively stronger bond between insulation and concrete. In addition, the ultimate strength of the test results was compared to the analytical prediction with the mechanical properties of only GRFP grids. The specimens with EPS insulation presented higher strength-based composite action than the ones with XPS insulation.

## 1. Introduction

Insulated concrete sandwich wall panels (SWPs) generally consist of the inner/outer concrete wythes and the insulation between the concrete wythes. Various types of insulated concrete SWP systems have been developed to increase both the thermal and structural efficiency. In line with the increasingly growing demand for energy-efficient buildings throughout the world, insulated concrete SWP systems have been drawing more attention. These systems have been applied to various building structures, such as residential and office buildings, cold storages, and industrial buildings. They have been more commonly used for the exterior wall, but they have also been used for the interior wall. There are various insulation materials, including fiberglass, rock wool, and polystyrene. The extruded polystyrene (XPS) and expanded polystyrene (EPS) foams are most commonly used for the insulated concrete SWP systems because they have high thermal performance and workability. Their energy-saving effects are higher than those of fiberglass under the same environmental conditions [1]. Moreover, their construction cost is lower than that of rock wool when the same thermal performance is secured [2].

Insulated concrete SWPs are categorized into a non-composite wall panel in which the inner/outer concrete wythes behave independently, a composite wall panel where an integrated wall panel behaves as a single member, and a partial composite wall panel depending on the degree of composite action. The inner and outer concrete wythes of the insulated concrete SWP are interlinked with various shear connectors, which are made from concrete, steel or fiber-reinforced plastic (FRP), including carbon, glass, and aramid fiber composites.

The difference in composite action among the insulated concrete SWPs depends on the degree of shear flow capacity, which is determined by the shear connector’s material and the adhesive bond between the insulation and the concrete wythes. Pessiki and MIynarczyk [3] evaluated the effect of the steel M-tie shear connector and solid concrete rib on the composite action. The effect of the steel M-tie shear connector that contributed to the composite action was relatively lower than that of the solid concrete rip. Various continuous shear connectors, including the steel-truss-shaped shear connectors, were reported to improve the composite action [4,5,6]. Also, a three-wythe insulated concrete SWP with a solid concrete rib showed nearly composite action [7].

Unlike the previous studies that employed metal or concrete shear connectors, Salmon *et al.* [8] introduced a FRP shear connector. The application of FRP to the building envelope system made it possible to obtain a higher thermal insulation effect compared to that obtained by concrete or metal shear connectors. Woltman *et al.* [9] performed push-off experimental tests on SWPs with bar-type glass-fiber-reinforced polymer (GFRP) and polypropylene shear connectors. The specimen with a GFRP shear connector showed higher strength and stiffness than one of the polypropylene connectors did, and the diameter of the shear connector did not have a significant effect on the shear strength of the SWP. Moreover, in another experimental test on four-point bending about SWP with the same type of GFRP shear connector [10], the flexural strength of the panel was 80% of the theoretical strength of the fully composite panel. In 2010, a Nebraska University research team conducted a study for the purpose of developing an SWP system with glass fiber NU-tie [11]. According to the study, NU-tie had a significant effect on the flexural strength and stiffness, and was able to achieve complete composite behavior. Pantelides *et al.* [12] evaluated the structural performance of SWPs using carbon-fiber-reinforced polymer (CFRP) shells connectors. They exhibited a quite good shear transmitting capacity, but there was some difficulty in producing insulated concrete SWPs. In another application of CFRP materials [13,14,15], the SWPs reinforced with grid-type CFRP shear connectors brought out an almost full-composite action of SWP, but although the design method for insulated concrete SWPs using metal or concrete core as shear connectors was recently presented at the Precast/Prestressed Concrete Institute (PCI) Committee [16], the study of insulated concrete SWP systems using FRP materials as shear connectors is still in progress, and there is room for improving the structural performance of SWP systems as well as of shear connectors.

**Figure 1:**
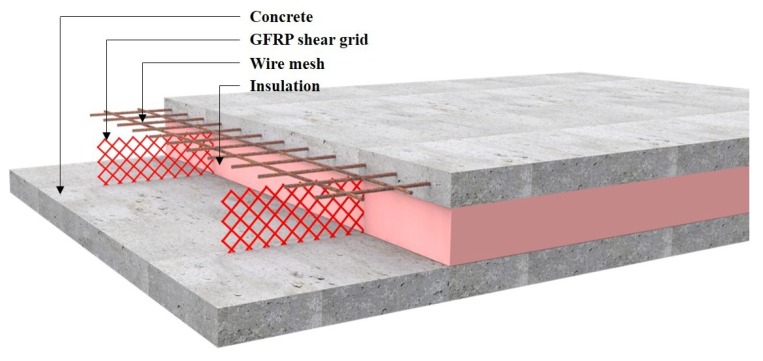
Insulated concrete sandwich wall panel reinforced with glass-fiber-reinforced polymer (GFRP) shear grids.

In this research, a novel insulated concrete SWP system, as seen in Figure 1, was developed to improve both the structural and thermal performance. This work focused on investigating the structural behavior of the proposed insulated concrete SWPs reinforced with GFRP shear grids. The degree of composite behavior in terms of strength and the effects of the different insulation types were examined using a full-scale experimental testing program. In addition, the analytical prediction of the flexural strength was compared with the experimental test results.

## 2. Materials and Experimental Program

### 2.1. GFRP Shear Grid for Insulated Concrete SWPs

A shear connector for insulated concrete SWPs should play the role of enhancing the composite action and reducing the energy loss by thermal bridge. In the construction industry, where traditional concrete and steel have dominated for the last 160 years, FRP has been attracting attention mainly because of its relatively higher tensile strength compared to the traditional steel material, and its low conductivity, light weight, and anticorrosive advantage. As a shear connector for an insulated concrete SWP system, the weight of FRP is only one fourth that of steel thanks to the former’s low specific gravity (1.4–2.0) while its tensile strength is 2 to 10 times that of steel, as seen in Table 1. The thermal conductivity of GFRP is only 1/200 that of steel, which enables the prevention of the occurrence of a thermal bridge or of condensation in the existing system by a steel stud penetrating the insulation layer.

**Table 1:** Material properties of the shear connectors [17,18,19].

Material	Strength, MPa	Density, kg/m^3^	Thermal conductivity, W/m·K
GFRP	482–2410	1800	0.3
Steel	240–689	7850	60
Aluminum	210	2700	191
Concrete	28–56	2300	2.1
EPS	0.07–0.26	20–30	0.032
XPS	0.15–0.70	28–32	0.028

**Figure 2:**
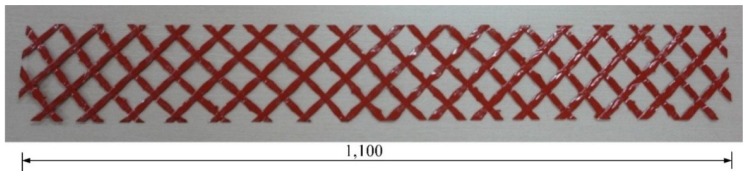
GFRP grid as a shear connector.

The GFRP grid shear connector consists of vertical and horizontal strands, as seen in Figure 2, and the tensile strength in the crossing direction should be equal. In this study, maintaining the straightness and equal tensile strength in the right-angle crossing direction was achieved by fixing the cross-section of the vertical and horizontal directions. The tensile strength of the glass fiber that was used for the GFRP composite was 1012 MPa. The glass fiber was coated with epoxy resin for the GFRP shear grid. Both the vertical and horizontal strands of the grids contained three roving 4400 TEX. The tensile strength of the strands was measured to average 6.3 kN, with a standard deviation of 0.39. The tensile modulus of elasticity of the strands was approximately 80 GPa. The minimal interval of the grid strands was set at 35 mm × 35 mm to maintain an about 57.9 kN/m shear flow capacity of a shear grid. (The details will be shown in Section 3.4).

### 2.2. Test Specimen

**Figure 3:**
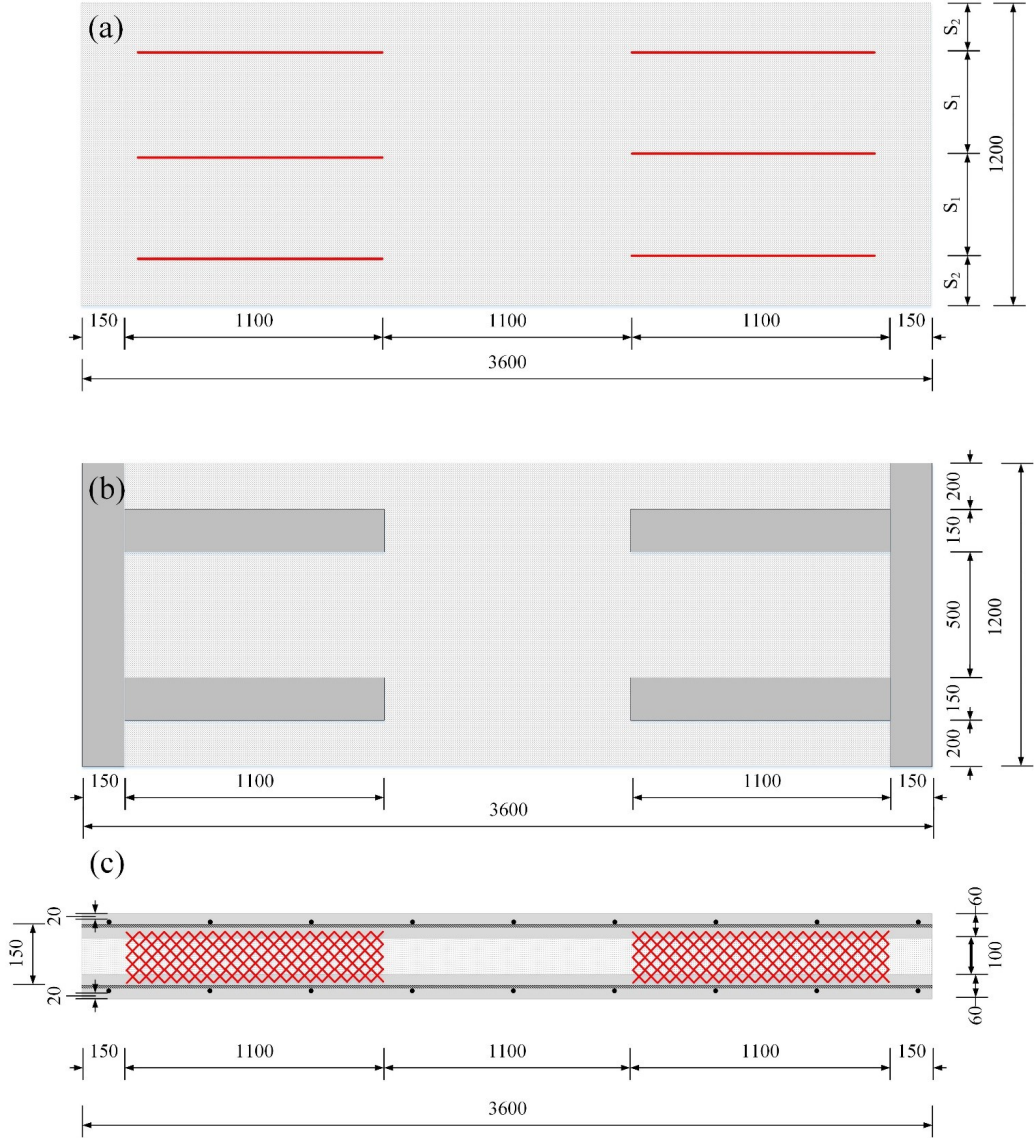
Dimensions of the specimens: (**a**) typical plan; (**b**) XPSC plan; (**c**) section.

As seen in Table 2, there were two groups of specimens depending on the type of insulation (XPS foam, EPS foam). The typical dimensions of the specimens are shown in Figure 3, and the GFRP shear grids were arranged in uniform intervals on both shear spans. The disposition of the GFRP shear grids is shown in Figure 3a, with two parameters: S_1_ and S_2_. S_1_ is the spacing distance between the GFRP shear grids, and S_2_ is the distance between the GFRP shear grids and the edges of the specimens. The values of S_1_ and S_2_ are presented in Table 2. The two concrete wythes of the XPSC specimen were connected with the concrete ribs (wth = 150 mm) for the complete composite behavior test, as seen in Figure 3b. XPS0 and EPS0 without any GFRP grid were fabricated to transfer the in-plane shear force through adhesion between the insulation and the concrete and the shear resistance only of the insulation. XPS1-3 and EPS1-4 were fabricated using one to four shear grids, which were used on both shear spans so that the in-plane shear force could be transferred not only through adhesion between the concrete and the insulation but also through the truss action of the GFRP grids. The thickness of the inner/outer concrete panel of all the specimens was 60 mm. The thickness of the insulation was determined to be 100 mm based on the thermal conductivity of insulation and building energy requirement for external walls in South Korea [20]. At the testing time, the concrete compressive strength was 45 MPa for the first group and 38 MPa for the second group. All of the specimens were reinforced with a D7 wire mesh for flexural reinforcement. The yielding and tensile strengths were 534 and 634 MPa, respectively. In this experimental program, some specimens had an uneven concrete thickness in the panel end, but this might not have had any effect on the flexural behavior of the specimens because the uneven parts were outside the support point.

**Table 2:** Test specimen matrix.

No.	Specimens	Label	Insulation (thickness, mm)	No. of Grid (or Rib)	GFRP strands	Wire mesh	S_e_ (mm)	S_2_ (mm)
1	1st group: Extruded polystyrene (XPS)	XPS0	XPS 100	0	4400TEX 3 strands	D7.0 @100	-	-
2		XPS1		1			-	600
3		XPS2		2			600	300
4		XPS3		3			400	200
5		XPSC		(2)			500	200
6	2nd group: Expanded polystyrene (EPS)	EPS0	EPS 100	0	4400TEX 3 strands	D7.0 @100	-	-
7		EPS1		1			-	600
8		EPS2		2			600	300
9		EPS3		3			400	200
10		EPS4		4			300	150

### 2.3. Test Equipment

**Figure 4:**
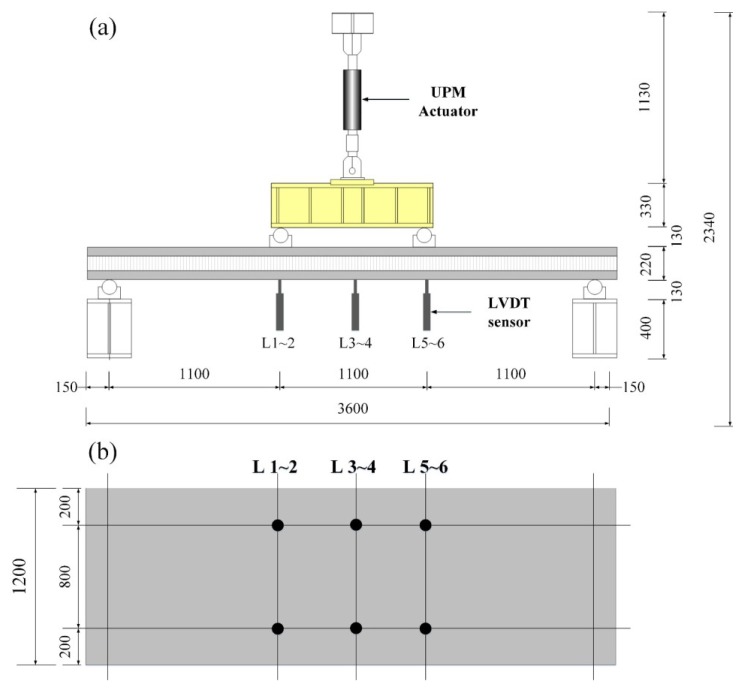
Test setup and measurement installation: (**a**) test setup; (**b**) linear variable differential transformers (LVDT) installation.

The specimen was set in the four-point loading condition, as seen in Figure 4a, to test the flexural performance of the developed insulated concrete SWP system. The displacement control scheme was used in loading with a 2000 kN actuator. Six linear variable differential transformers (LVDTs) were installed to measure the deflected shapes of the specimens, as seen in Figure 4b. The mid-span deflection of the specimens was taken with the average measurement values from the two LVDTs in the mid-span.

## 3. Results and Discussion

### 3.1. Specimen with Extruded Polystyrene (XPS) Foam

**Figure 5:**
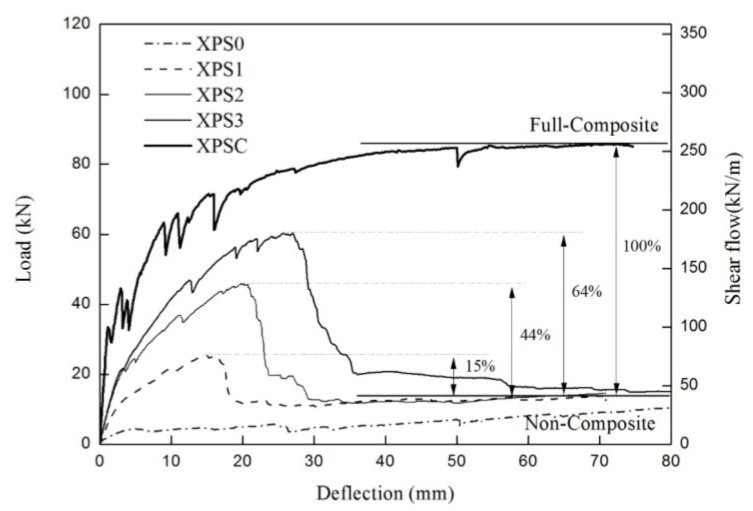
Load-deflection curve of the specimen using extruded polystyrene (XPS) insulation.

**Figure 6:**
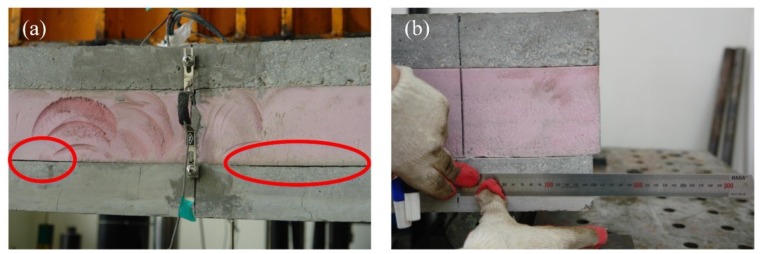
Failure modes of the specimen using XPS insulation: (**a**) bond failure of XPS insulation; (**b**) slip between the concrete and the XPS insulation.

Figure 5 shows the load-deflection curves for all the specimens in the first group. In the XPS0 specimen, the insulation was debonded from a concrete wythe during handling because of the low bond strength between the concrete and the insulation. This damage resulted in a lower-than-expected flexural strength, which reflected the bond effect between the insulation and the concrete. At the initial loading stage for the XPS1-3 specimens, the in-plain shear forces were resisted by the shear strength from the GFRP grid(s) at each shear span as well as by the interface adhesion between the XPS foam and the concrete. When the deflection at the mid-span reached 3–4 mm, flexural crack began to occur in the bottom concrete wythe around the mid-span, and the slope of the load-displacement curve was gradually reduced. As the load increased, the XPS foam got debonded from the concrete wythes, as seen in Figure 6, and slip occurred at the interface, especially in the cracked region. The specimen exhibited some degree of composite action until the maximum load reached 25–60 kN depending on the number of GFRP grids. Finally, the GFRP grids at either shear span were entirely ruptured, followed by a significant load decrease. Then two concrete wythes showed non-composite behavior against flexural loading, and a significant slip was observed between them. The XPSC specimen was designed to demonstrate the complete composite behavior, and showed such behavior at the 80 kN or higher load. In the XPSC specimen, the concrete ribs have not failed over the maximum load even after the wire mesh yielded, and the composite behavior of the two concrete wythes had been maintained until the displacement reached 75 mm. Assuming that the maximum load of XPSC was full-composite, and that the mean loads of the specimens (XPS1-3) after the rupture of the GFRP grids were non-composite, the degree of composite action was analyzed in terms of strength, using Equation (1). In Equation (1), P_full_ is the experimental maximum load with full-composite action, P_non_ is the experimental maximum load with non-composite action, and P_e_ is the experimental maximum load of the test specimen. As indicated in Figure 6, the degrees of composite action of the XPS1-3 specimens were 15%, 44%, and 64% of the full-composite action, respectively.

(1) $$\kappa =\frac{\left(P_{e}-P_{\mathrm{non}}\right)}{\left(P_{\mathrm{full}}-P_{\mathrm{non}}\right)}\times 100\left(\%\right)$$

Table 3 presents the stiffness and strength at the three response stages. In the initial stage, there was no crack, and thus, the elastic behavior was maintained. In the second stage, flexural cracks were initiated, and the XPS foam was partially separated from the concrete wythes. Most of the shear forces were transmitted by the GFRP grids at this stage, and the number of grids made an obvious difference especially in the strength. In the third stage, the stiffness dropped, and the loading capacity reached the maximum load, where the GFRP strands of the grids got gradually ruptured. As a result, the flexural strength of the specimens with XPS foam increased in proportion to the number of GFRP grids.

**Table 3:** Stiffness and strength in the three response stages.

Label	Initial		Second		Third		Failure Mode
	Stiffness (kN/m)	Strength (kN)	Stiffness (kN/m)	Strength (kN)	Stiffness (kN/m)	Strength (kN)	
XPS1	4.55	10	1.58	20	0.07	25	Bond failure, shear grid rupture
XPS2	7.03	20	1.98	37	0.33	46	Bond failure, shear grid rupture
XPS3	7.11	21	2.07	46	0.15	60	Bond failure, shear grid rupture
XPSC	30.46	34	7.95	66	0.33	86	Steel fracture
EPS0	2.94	31	1.53	52	0.16	71	Insulation shear failure
EPS1	3.58	33	1.49	59	0.26	74	Insulation shear failure, shear grid rupture
EPS2	3.66	34	1.24	62	0.32	72	Insulation shear failure, shear grid rupture
EPS3	3.46	39	1.45	65	0.23	77	Insulation shear failure, shear grid rupture
EPS4	4.68	44	1.47	71	0.28	84	Steel fracture

### 3.2. Specimen with Expanded Polystyrene (EPS) Foam

**Figure 7:**
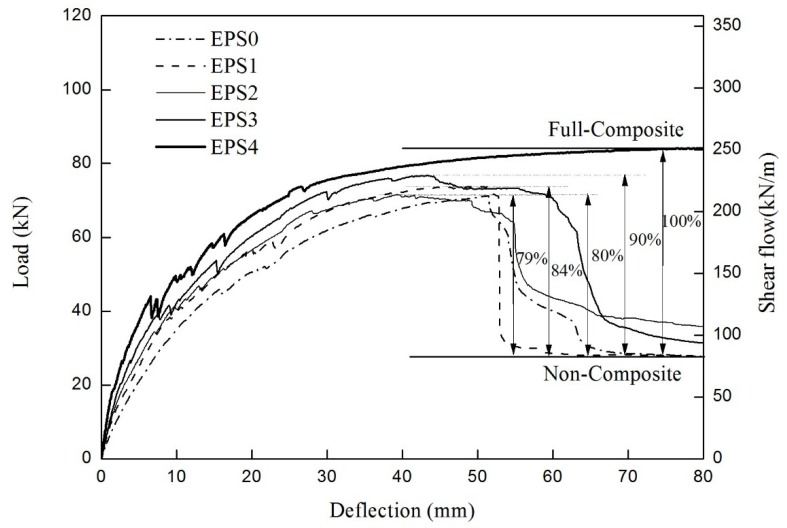
Load-deflection curve of the specimen using expanded polystyrene (EPS) insulation.

**Figure 8:**
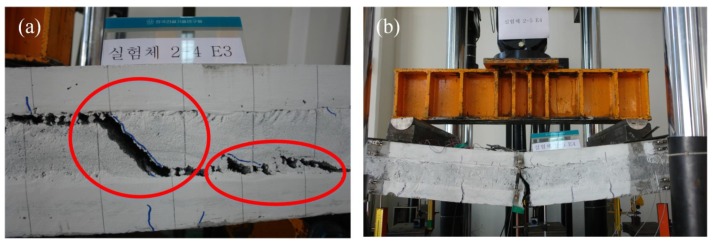
Failure modes of the specimen using EPS insulation: (**a**) shear failure of EPS insulation; (**b**) reinforcement rupture in the EPS4 specimen.

As seen in Figure 7, all the specimens reached their peak at 71–85 kN. The dependence on the number of grids was much weaker than that of the specimen with XPS foam. No interface failure between the concrete and the insulation was observed until the strands of the GFRP grids got fractured. As this strong adhesion between concrete and insulation might have a great effect on resisting the in-plane shear force, the two concrete wythes behaved near full-composite action against the applied load. When the GFRP grids ruptured, the EPS insulation was torn in the 45° direction in either shear span, as shown in Figure 8a, resulting in a significant decrease in strength. The concrete wythes began to be separated at the torn insulation surface, and they resisted the bending moment independently as non-composite behavior, followed by a significant slip. As for the EPS4 specimen, the GFRP grids did not entirely fail, and the two concrete wythes maintained full-composite action until the flexural reinforcement (wire mesh) in the bottom concrete wythe was fractured. As shown in Table 3, the stiffness and strength values in all the three stages fell within a small range; as such, the number of grids did not govern the flexural behavior of the ESP specimens in the second group. Assuming that the EPS4 specimen was full-composite, the degree of composite action was analyzed in terms of the load, and as seen in Figure 7, it was about 79%–90% of the full-composite behavior regardless of the number of GFRP grids. Therefore, in the specimens with EPS foam, the initial stiffness was somewhat increased in proportion to the number of GFRP grids, but the relationship between the number of GFRP grids and the flexural strength was not clear.

For all the XPS and EPS specimens, except for EPS4, the GFRP shear grids ruptured at the maximum load, and then the loading capacities rapidly dropped. At this maximum load, the composite behavior of the two concrete wythes were converted to independent (non-composite) behavior of each concrete wythe because the shear grids were ruptured. It was concluded that the maximum loads were governed mainly by the GFRP capacity. In addition, the concrete compressive strength could be an important factor influencing the pullout strength of the GFRP shear grids. In the preliminary test, pullout of the GFRP shear grids did not occur when the anchorage length of the GFRP shear grids was more than 20 mm, with f’c = 35 MPa or less. It was ensured that the anchorage length of the GFRP grids in this test was about 25 mm, and thus, no pullout failure occurred during the test. Therefore, as the concrete compressive strength was high enough to prevent the pullout failure of GFRP, the concrete compressive strength did not significantly affect the maximum load, which was governed by the capacity of the GFRP shear connectors.

### 3.3. Effects of the Insulation Type

**Figure 9:**
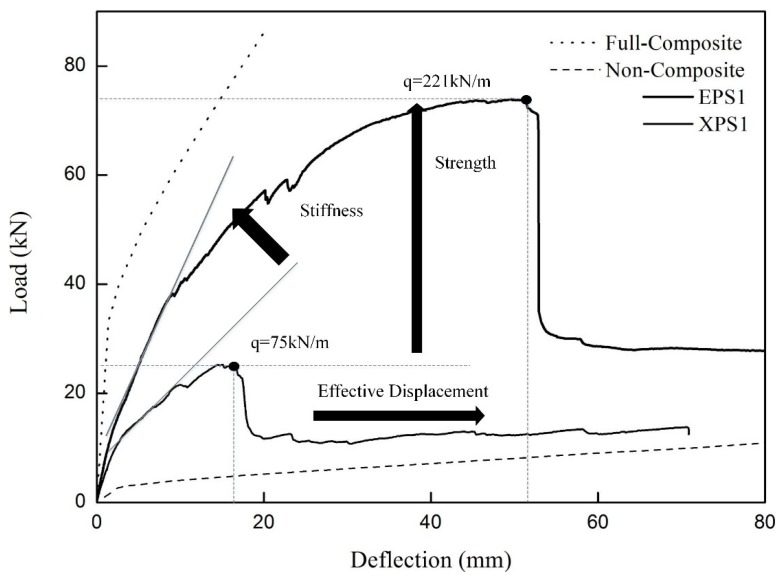
Comparison of the load-deflection curves depending on the insulation type.

The load-deflection behaviors of the XPS and EPS specimens, where one GFRP grid on each shear span was used, are compared in Figure 9. In the case of the XPS specimen, the flexural crack in the concrete wythes initiated interface failure between the concrete and the insulation. The early separation of the concrete and the insulation resulted from the significantly low bond strength. Thus, the GFRP grids should transfer almost the full horizontal in-plane shear force between the concrete and the insulation until they are ruptured. On the other hand, the EPS specimen underwent no interface failure between the insulation and the concrete even after the occurrence of a major flexural crack. Thus, the shear strength of the EPS foam and the truss mechanism of the GFRP grids resisted the in-plane shear force together. For such reason, the flexural strength of the EPS specimen was higher than that of the XPS specimen reinforced with the same number of GFRP grids. In terms of stiffness, the significantly lower stiffness of the XPS specimen was monitored due to the gradual interface failure after the occurrence of a flexural crack in the second stage. In addition, the adhesion between the EPS foams and the concrete allowed the improvement of a synergetic resistance combining the grids and the insulation, and enhanced the deformation capacity until the EPS foams failed in the diagonal shear. Such behavior was also monitored from the specimens with two and three GFRP grids on both shear spans. 

Table 4 summarizes the moment and deformation capacities. The deformability was defined as a post-cracking deformable capacity by calculating the deflection difference between the ultimate state and the crack initiation. The moment capacities of the EPS specimens were 1.7–3.3 times larger than those of the XPS specimens at the crack initiation, and 1.3–2.9 times larger at the ultimate state. The deformabilities of the XPS specimens were improved from 12.5 to 23.9 mm as the number of GFRP shear grids increased, while all the EPS specimens exhibited a comparatively very large deformability of 46–52 mm if including the ductile behavior after the ultimate deflections. The enhanced deformation capacity of the EPS specimens is shown in stage 3, where the stiffness decreased rapidly and the loading capacity plateaued as the GFRP strands of the grids got gradually ruptured and as the steel wire mesh yielded.

**Figure 10:**
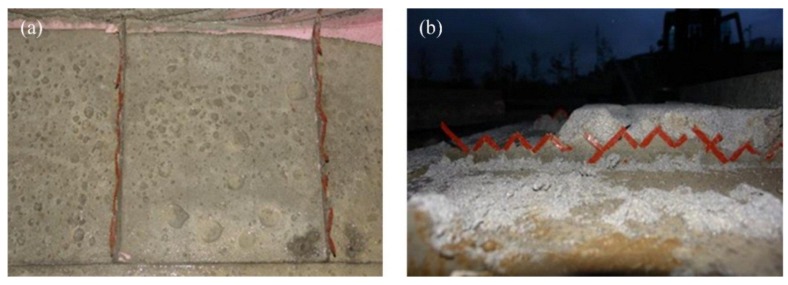
Bond failure surfaces of the concrete wythes: (**a**) XPS specimen; and (**b**) EPS specimen.

Figure 10 shows the foam-removed concrete surface with ruptured grids rather than pullout failure of the grids. The concrete surface in (a) the XPS specimen looked smooth, but many fragments of the EPS foam remained stuck with the concrete surface in (b) the EPS specimen. As a result, the degree of composite action in the insulated concrete SWP system was significantly influenced by the type of insulation, which had different adhesion property to concrete surface.

**Table 4:** Comparison of the moment and deformation capacities.

Label	Crack Initiation		Ultimate State		Deformability (mm)
	Moment (kN/m)	Deflection (mm)	Moment (kN/m)	Deflection (mm)	
XPS1	5.5	2.1	13.8	14.7	12.6
EPS1	18.2	7.2	40.7	48.3	41.1(45.5)
XPS2	11	2.8	25.3	20.2	17.4
EPS2	18.7	6.6	39.6	39.4	32.8(48.3)
XPS3	11.6	2.9	33.0	26.9	23.9
EPS3	21.5	6.5	42.4	42.4	35.9(52.7)

The deformability of each specimen was calculated by subtracting the deflection at the crack initiation from the deflection at the ultimate state. The bracketed values present the extended deformability, which includes the ductile deformation until before the load capacity dropped abruptly due to the GFRP grid rupture.

### 3.4. Analytical Prediction

**Figure 11:**
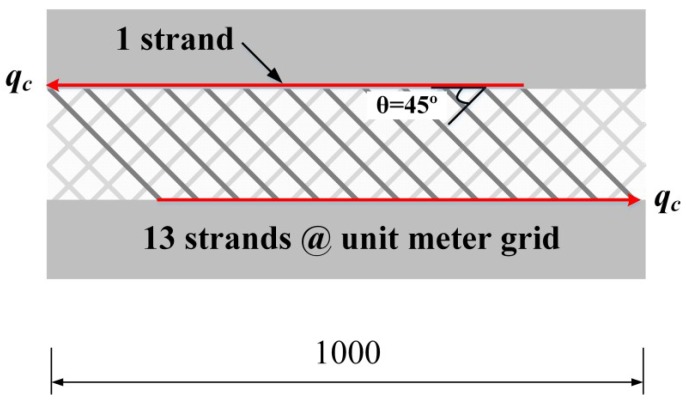
Shear flow strength based on the tensile strength of the GFRP strands.

**Figure 12:**
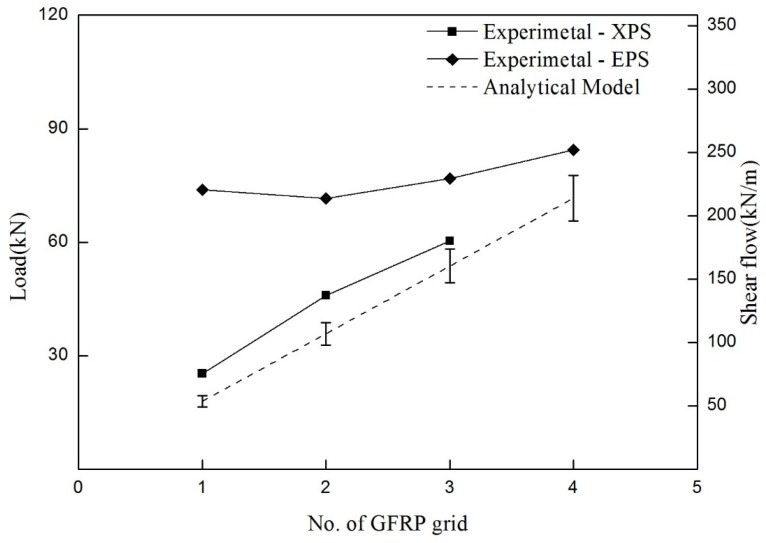
Maximum loads of the experimental test and analytical model.

The flexural capacity of the insulated concrete SWP with shear grids can be predicted using the shear flow strength of the GFRP shear grids in Equation (2).

(2) $$\mathrm{maximum}\;P=2V,V=\frac{q_{c}\times I}{Q}$$

where $q_{c}=NoGS\times f_{st}\times cos\;\theta$, *P* and *V* are the maximum load and the maximum shear force in the four-point bending system. *I* and *Q* are the moment of inertia and the first moment of area at the interface between the insulation and the concrete, assuming full-composite action. NoGS is defined as the number of active strands per unit meter grid, which was determined to be 13 for the shear grids used in this experimental program. The active strands refer to the strands whose two ends are embedded in the inner/outer concrete wythes, as seen in Figure 11. $f_{st}$ is the tensile strength of a GFRP strand of 6.3 kN (standard deviation: 0.39), and the shear flow strength of $q_{c}$ is approximately 57.9 kN/m. Assuming a full-composite section, *I* and *Q* were computed as 5,760,000 mm^3^ and 964,800,000 mm^4^, respectively. In the case of an SWP system reinforced with a shear grid per shear span, the SWP system is expected to resist a 19.4 kN load, and the expected maximum load for the four shear grids per shear span was computed as 77.6. To ensure the GFRP grid failure before flexural failure, a 7 mm-diameter wire mesh was selected because the specimen with the 7 mm-diameter wire mesh provided a more than 80 kN loading capacity. As only the shear connector is considered, without adhesion of the concrete and insulation, the expected load may be a lower limit (low bound). The expected maximum load increases linearly, as shown in Figure 12, when the number of shear grids increases.

The shear flow capacities of the XPS specimens were improved by about 15 kN/m from the analytical prediction considering only the shear grids. This was approximately 20% of the shear flow strength of the unit grid. On the other hand, in the group of EPS specimens, the shear flow strength measured in the experimental test was much larger than that predicted analytically. As the adhesion characteristics seemed to govern the load capacity rather than the number of grids, it was very complex to quantify the insulation effects on the analytical prediction. It was noted that the shear flow strength for the EPS4 specimen might be underestimated because the EPS4 specimen exhibited the steel wire mesh fracture before the entire rupture of the GFRP shear grids. For further study, strength enhancement according to the type of insulation should be taken into account in the analytical prediction of the flexural strength.

## 4. Conclusions

The purpose of this study was to develop a novel insulated concrete SWP system using GFRP grid shear connectors. A full-scale experimental program was performed to verify the structural performance of the insulated concrete SWPs. In addition, the analytical prediction was compared to the experiment results. Basically, the SWPs reinforced with GFRP grids as shear connectors hold an enhanced flexural capacity by developing a high degree of composite action. The other test results and findings from this study are outlined as follows.

The degree of composite action and the flexural strength of the specimens with extruded polystyrene (XPS) insulation were increased in proportion to the number of GFRP grids, but the contribution of the adhesion between the concrete and the insulation was minor because of the smooth surface of the XPS foam. As for the specimens with expanded polystyrene (EPS) insulation, the in-plane shear strength was mainly dependent on the adhesion between the concrete and the insulation, and thus, the flexural strength was little affected by the number of shear grids. The maximum flexural strength was mostly dependent on the in-plane shear resistance provided by the bond between the insulation and the concrete, and a full-composite behavior was achieved when four or more GFRP grids were used in this experimental program.

The strength-based degree of composite action in the insulated concrete SWP system was significantly influenced by the type of insulation, which had different adhesion properties to the concrete surface. The in-plane shear strength of the insulated concrete SWPs with EPS foam was much higher than that of the specimen with XPS foam because of the high bond capacity of EPS foam to concrete panels. Accordingly, it is a very good option to improve the interface adhesion between concrete and insulation when applying XPS foam to insulated concrete SWP systems.
